# Age modifies the genotype-phenotype relationship for the bitter receptor *TAS2R38*

**DOI:** 10.1186/1471-2156-11-60

**Published:** 2010-07-01

**Authors:** Julie A Mennella, M Yanina Pepino, Fujiko F Duke, Danielle R Reed

**Affiliations:** 1 Monell Chemical Senses Center, 3500 Market St, Philadelphia PA, 19104, USA

## Abstract

**Background:**

The purpose of this study was to investigate the effect of *TAS2R38 *haplotypes and age on human bitter taste perception.

**Results:**

Children (3 to 10 yrs), adolescents (11 to 19 yrs) and adults (mostly mothers, 20 to 55 yrs (N = 980) were measured for bitter taste thresholds for 6-n-propylthiouracil (PROP) and genotyped for three polymorphisms of the AS2R38 gene (A49P, V262A, I296V). Subjects were grouped by haplotype and age, as well as sex and race/ethnicity, and compared for PROP thresholds. Subjects with the same haplotype were similar in bitter threshold regardless of race/ethnicity (all ages) or sex (children and adolescents; all p-values  > 0.05) but age was a modifier of the genotype-phenotype relationship.  Specifically, AVI/PAV heterozygous children could perceive a bitter taste at lower PROP concentrations than could heterozygous adults, with the thresholds of heterozygous adolescents being intermediate (p < 0.001).  Similar age effects were not observed for subjects with the PAV/PAV or AVI/AVI homozygous haplotypes (p > 0.05) perhaps because there is less variation in taste perception among these homozygotes.

**Conclusions:**

These data imply that the change in PROP bitter sensitivity which occurs over the lifespan (from bitter sensitive to less so) is more common in people with a particular haplotype combination, i.e., AVI/PAV heterozygotes.

## Background

The experience of bitterness occurs after certain chemicals contact taste receptors located in cells on the surface of the tongue. Some investigators hypothesize that this sense provides information so that people do not ingest bitter-tasting toxic chemicals [1]. Potent poisons are found in many plants (e.g., like ricin and castor beans) which render them inedible [2]. However for many other plants, the potency or amount of toxin is low enough so that even though some (e.g., turnips or cabbage) might taste bitter, they can be eaten with fewer consequences [3]. However this poison detection system is not perfect because not everyone perceives the intensity of a fixed bitter stimulus in the same way [4,5]. The classic example of individual differences in taste sensitivity is for phenylthiocarbamide (PTC) and the related chemical propylthiouracil (PROP) [6]. Some people can detect these compounds at low concentrations, whereas others need much higher concentrations or cannot detect them at all [7,8]. Early family studies suggested this bimodality in taste response was due to alleles in a single gene [8,9]. Based on clues from many population studies, investigators predicted the minor allele frequency of the hypothetical locus would be high because bitter insensitivity to these compounds was common. They also predicted that the allele frequency would vary between human populations [6,10]. These predictions later proved to be accurate, with qualifications [11].

The gene which accounts for this taste trait is *TAS2R38*, a member of the family of taste receptors that respond to bitter stimuli [12-16] and the milestones of this discovery have recently been summarized [17]. One qualification to previous predictions mentioned above was that the molecular characterization of this genetic locus revealed three sites of genetic variation (A49P, V262A, and I296V) which are found in two common (AVI and PAV), two less common (AAI and AAV) and two rare haplotypes (PVI and PAI) [11,18-21]. Two haplotypes have not been observed in any subjects tested to date, AVV and PVV. As foreseen by earlier investigators, the frequency of the two common haplotypes varies by race/ethnicity [11,18,19]. Thus heterozygosity is common and may be due to balancing natural selection as a result of as yet unknown beneficial properties of the minor allele [18,22]. Another feature of this locus is that while homozygosity is associated with extremes of PROP threshold, heterozygosity is associated with a wide range of taste ability [12]. For a translation of genotype, haplotype and diplotype and their effects on taste perception for PROP, see Table 1.

**Table 1:** Translation of *TAS2R38 *genotypes/haplotypes/diplotypes to PROP taste perception.

+49	+262	+296	Haplotype	Diplotype	PROP Taste perception
A	V	I	AVI	AVI/AVI	Nontaster
P	A	V	PAV	PAV/PAV	Taster

Adapted from [11]. The terms 'taster' and 'nontaster' are used for convenience because there is a range of perceptual ability within each category. +49, +262 and +296 refer to amino acid locations in the *TAS2R38 *protein. The single letter notations refer to amino acids, e.g., A = Alanine.

Investigators have observed that younger subjects are more sensitive than older subjects to the bitterness of PROP or PTC, with some suggesting that age modifies the genotype-phenotype relationship [8,23-29]. Since allele frequencies do not differ between children and adults, the explanation for age-related differences in bitter perception must lie elsewhere. Previous investigators noted that people who were less sensitive to this class of bitter compounds seemed to lose their sensitivity faster as they got older, concluding that gene penetrance might differ by age and genotype [30,31]. To try to understand this issue, in an earlier study, we assessed children and their mothers (N = 257) for the first genetic variant site (A49P) of the *TAS2R38 *gene and measured their PROP thresholds. We found that children who were heterozygous (AP) were more bitter sensitive than adults of the same genotype but that AA or PP homozygous children did not differ from adults of the same genotypes [32].

There were three unanswered questions that arose from this study. First, only the first variant site was typed. Thus, subjects who were homozygous for the A49 allele could have one of several haplotypes: (A)VI, (A)AI or (A)AV, and therefore some people classed as homozygous for the first allele (AA) would be heterozygous for the other alleles if the remaining haplotype was considered. This point is important because subjects with an AA haplotype have an intermediate phenotype [21]. Second, only children between the ages of 5 to 10 years and adults were studied so we could not determine when this change occurred. There were enough subjects to categorize people into three groups by genotype (AA, AP or PP at position +49) but too few to group by haplotype or diplotype. Therefore in the present study, we phenotyped a large (N = 980) and diverse group of children, adolescents and adults and genotyped them for three *TAS2R38 *alleles. These data now allow us to gauge the interaction between age and diplotype and the timing at which changes in gene penetrance for PROP bitter taste perception occurs.

## Results

### Subjects

Subjects who participated in research studies on taste and smell preferences during the years 2003-2007 were phenotyped for PROP threshold and genotyped for three alleles of the *TAS2R38 *gene. Included in this sample of 980 individuals were 448 children (241F/207M), 100 adolescents (55F/45M) and 432 adults (425F/7M). The majority of the adult subjects (N = 345) were the mothers of the children or adolescent participants. Children ranged in age from 3 to 10 years (mean 7 ± 2), adolescents from 11-19 years (mean 15 ± 2) and adults from 20 to 55 years (mean 34 ± 7). Race/ethnicity was assigned by maternal (or adult) report according to standard US Census categories. We used the term race/ethnicity in describing our groups because it represents both the genetic and cultural components of this sample [33]. These categories reflect the population of the urban setting from which it was drawn: Philadelphia, Pennsylvania, USA; 56% African-descent (Non-Hispanic; African-American), 29% Caucasian (Non-Hispanic; Caucasian) and 15% other groups (Mixed ancestry, Asian, or Hispanic). All testing procedures were approved by the Office of Regulatory Affairs at the University of Pennsylvania. Informed consent was obtained from each adult and assent was obtained from each child who was 7 years of age or older.

### Haplotypes and diplotypes

A breakdown of diplotype frequency by age and race/ethnicity is provided in Table 2. From all combinations of the three alleles, six out of the eight possible haplotypes and thirteen of the thirty-six possible diplotypes were observed. Two haplotypes accounted for over 84% of all haplotypes (AVI, 41.2%, nontaster and PAV, 43.1%, taster) whereas the remaining four haplotypes were rare (AAI, 12.2% and AAV, 3.3%) or extremely rare (PAI, <1% and PVI, <1%). The two remaining possible combinations, AVV and PVV, were not observed in this sample.

**Table 2:** Distribution of *TAS2R38 *diplotype by age and race/ethnicity

Race	Age group	AVI/AVI	AAI/AVI	AAV/AVI	AAV/AAI	AAI/AAI	AAV/AAV	AVI/PAV	AAI/PAV	AAV/PAV	AAI/PAI	PAV/PVI	PAV/PAI	PAV/PAV	Total
Caucasian	Children	**32**	2	8	0	0	0	**60**	1	10	0	0	0	**21**	134
	Adolescents	**2**	0	0	0	0	0	**6**	0	1	0	0	0	**3**	12
	Adults	**37**	1	5	1	0	0	**60**	1	6	0	0	0	**25**	136
	Total	**71**	3	13	1	0	0	**126**	2	17	0	0	0	**49**	282
	Percent	**25**	1	5	<1	0	0	**45**	<1	6	0	0	0	**18**	
															
African-American	Children	**35**	26	2	0	7	0	**73**	39	5	0	0	1	**44**	232
	Adolescents	**8**	10	0	1	6	0	**22**	15	1	0	1	0	**7**	71
	Adults	**31**	29	4	0	9	0	**86**	37	10	1	1	0	**37**	245
	Total	**74**	65	6	1	22	0	**181**	91	16	1	2	1	**88**	548
	Percent	**14**	12	1	<1	4	0	**33**	17	2	<1	<1	<1	**16**	
															
Other	Children	**14**	8	3	0	0	1	**28**	9	2	0	0	0	**17**	82
	Adolescents	**3**	2	0	0	1	0	**5**	2	1	0	0	0	**3**	17
	Adults	**10**	6	0	1	0	0	**18**	2	1	0	0	0	**13**	51
	Total	**27**	16	3	1	1	1	**51**	13	4	0	0	0	**33**	150
	Percent	**18**	11	2	<1	<1	<1	**34**	9	3	0	0	0	**22**	
															
All	Total	**172**	84	22	3	23	1	**358**	106	37	1	2	1	**170**	980
	Percent	**18**	9	2	<1	2	<1	**37**	11	4	<1	<1	<1	**17**	

The number of children, adolescents, and adults by race/ethnicity and diplotype. Other = includes Asian, Hispanic and Mixed ancestry. The most common diplotypes are in **bold **text.

We refer to AVI as the 'nontaster' haplotype and PAV as the 'taster' haplotype for convenience, acknowledging that this use of terminology is an over-simplification. To make one further point of terminology, subjects with two copies of the taster or nontaster haplotype are referred to as having the taster or nontaster diplotype, respectively. The most frequent diplotype was the combination of the two most frequent haplotypes and these heterozygous subjects accounted for 37% of all subjects, followed in frequency by the homozygous subjects: 18% (AVI/AVI, nontaster) and 17% (PAV/PAV, taster). The remaining diplotypes were combinations of one rare and one common haplotype, except for three subjects, each of whom had a different combination of rare haplotypes.

### Age effects are most apparent in heterozygous subjects

A multinomial logistic regression was conducted and the results indicated a main effect of diplotype Χ^2^_(6) _= 323.62, p < 0.0000, a trend toward an effect of age Χ^2^_(6) _= 12.29, p = 0.0557 and a significant diplotype by age interaction [Χ^2^_(9) _= 22.62, p = 0.0071]. When half of the heterozygous subjects were included in the analysis, to equate group sizes, the results were similar: a main effect of diplotype, Χ^2^_(6) _= 271.71, p < 0.0000, no effect of age Χ^2^_(6) _= 3.67, p = 0.7200 and a significant diplotype by age interaction [Χ^2^_(9) _= 19.36, p = 0.0223]. To analyze the effects of age, independent of familial relationship, we conducted a similar analysis on a sub-sample of 508 unrelated people with the three most common diplotypes (AVI/AVI, AVI/PAV and PAV/PAV) and two age categories (children and adults) as fixed factors. Among unrelated individuals, there was a main effect of age Χ^2^_(3) _= 8.1, p = 0.04; a main effect of diplotype, Χ^2^_(6) _= 180.3, p < 0.0000 and an age by diplotype interaction Χ^2^_(6) _= 17.1, p = 0.0070.

**Figure 1:**
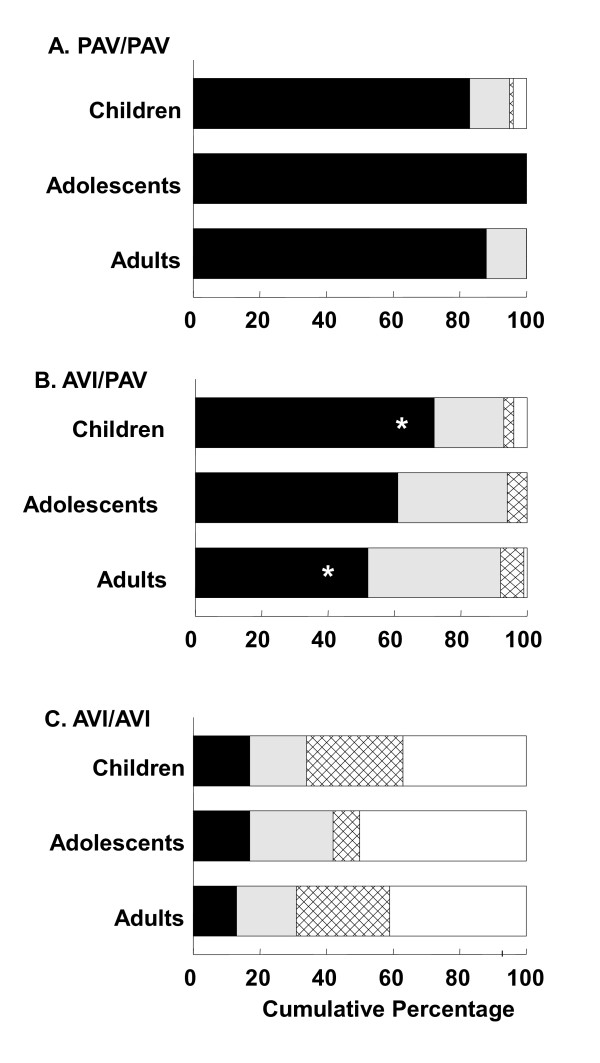
**Effect of age group on PROP sensitivity by common diplotypes**. The cumulative percentage of subjects with the following diplotypes (A) taster PAV/PAV, (B) heterozygous AVI/PAV, and (C) nontaster AVI/AVI who first reported a bitter taste when sampling 56 (black bars), 180 (grey bars), and 560 μM PROP (hatched bars) or who never reported a bitter taste when sampling each of these PROP solutions (white bars). In panel (B) there is an increase in the proportion of children who report a bitter taste for the 56 μmol/liter solution relative to adults. *denotes a significant difference by χ^2 ^partition.

To determine the nature of the diplotype by age interaction, groups were stratified by age group (children, adolescents and adults) within the most common diplotype groups (PAV/PAV, PAV/AVI, AVI/AVI) and percentages of subjects that could perceive bitterness at each concentration of PROP were compared. In a related analysis, we broadened the definition of heterozygotes to include subjects with one PAV haplotype and any other haplotype on the second chromosome (PAV/A**). As shown in Figure 1, age was a modifier of the genotype-phenotype relationship for heterozygous subjects. More heterozygous children perceived bitterness at the lowest concentrations than did adults with the same genotype, with adolescents intermediate between adults and children (AVI/PAV; omnibus, χ^2^_(4) _= 16.44, p = 0.0025; children and adolescents versus adults, χ^2^_(1) _= 12.86, p = 0.0003; children versus adolescents and adults, χ^2^_(1) _= 16.10, p < 0.0001). PAV/PAV or AVI/AVI homozygous children, adolescents and adults did not differ in PROP thresholds (all p-values > 0.05). These results were similar when the broader definition of heterozygotes was used (PAV/A**; omnibus χ^2^_(4) _= 16.90, p = 0.0020; children and adolescents versus adults, χ^2^_(1) _= 11.88, p = 0.0006; children versus adolescents and adults,  χ^2^_(1) _= 15.46, p = 0.0001).

### Race/ethnicity and sex do not affect genotype-phenotype relationships

To determine whether race/ethnicity had independent effects on PROP thresholds, we focused on people with four haplotypes (PAV, AVI, AAI and AAV) and excluded those with the two rarest haplotypes (PAI and PVI; N = 4) to meet the requirements of the statistical test. The frequencies of these four haplotypes differed between racial groups, specifically, the AAI haplotype was found more often in African-Americans whereas the AVI and AAV haplotypes were found more often in Caucasians (omnibus, χ^2^_(6) _= 123.10, p < 0.0000000; partition for AAI, χ^2^_(1) _= 105.02, p < 0.0000000; partition for AVI χ^2^_(1) _= 8.28, p = 0.00401; partition for AAV, χ^2^_(1) _= 8.86, p = 0.00292).

Next, we determined whether there were race/ethnicity effects on PROP thresholds within the most common diplotype groups (AVI/AVI, AVI/PAV and PAV/PAV). Other groups were excluded from this analysis, either because the contributing haplotypes were too rare for statistical comparisons or because the contributing haplotypes were specific to one racial group. We used a χ^2 ^test when possible, but in the case of the PAV/PAV group, a proportion test was used to compare the most sensitive tasters (who perceived a bitter taste from PROP at the lowest concentration) with all other taste groups. This step was necessary because so few subjects with a PAV/PAV diplotype were insensitive to PROP. With these details in mind, within a diplotype, we found no differences between African-American and Caucasian subjects in PROP sensitivity (all p-values > 0.05).

To determine whether sex had independent effects on PROP thresholds we focused on children and adolescents, groups with roughly equal numbers of boys and girls, because most of the people in the adult group were women (i.e., mothers). There were no sex differences in PROP thresholds in children and adolescents (χ^2^_(3) _= 5.52, p = 0.137).

## Discussion

The objective of this study followed from our previous study [32] and aimed to determine how age interacts with diplotype to affect PROP taste perception. While the association between *TAS2R38 *genotype and PROP taste sensitivity phenotype was resilient against effects of race/ethnicity (among all ages) and sex (in children in adolescents), age was a modifier of the association, especially among AVI/PAV heterozygotes. We found that children who were heterozygous for the common haplotypes were more sensitive to the bitterness of PROP than adults with the same diplotype, with adolescents intermediate. These findings are consistent with our earlier studies of children and their mothers which examined the effect of one *TAS2R38 *genotype (A49P) [32] as well as research of others who observed age-related changes in the perception of PTC and PROP [8,23-26,28,29]. The age effect was specific to diplotype and not detected among PAV/PAV homozygous taster or AVI/AVI homozygous nontaster subjects although this result might be partially explained by restricted range of phenotype of people with those homozygous diplotypes. Sample size differences which occur naturally between diplotype groups did not explain this effect because when groups were equated, the diplotype by age interaction was still evident. In addition, this result generalized to other heterozygous subjects because we observed it among subjects with rare heterozygous diplotypes (PAV/A**). Thus we conclude that these types of age-related changes in taste sensitivity are more pronounced in subjects with particular genotypes. This interaction between age and genotype is predicted to have a broad impact because many people in the population are heterozygous.

What causes the developmental shift in taste sensitivity among AVI/PAV heterozygotes is unknown. One explanation is that the age and diplotype interaction may be due to preferential allele expression, with children over-expressing the taster form rather than the nontaster form of the receptor early in life and then losing this tendency as they age. Adults heterozygous for the *TAS2R38 *gene do not express mRNA of each haplotype in a one-to-one proportion [12], so it is possible that heterozygous children might have a skewed expression pattern, perhaps over expressing the taster allele. If true, why this change would occur during adolescence is not known, but it may be triggered by signals that brain and body growth are complete [34]. The hypothesis that allelic expression could account for these phenotype differences is provocative, but necessarily speculative, because biopsy tissue of taste papillae of healthy children is not readily available for mRNA analysis. PROP bitterness is not entirely explained by alleles of the *TAS2R38 *gene [12,35-37] and these other modifiers (like taste papillae number) could be age-sensitive and more influential in heterozygous diplotypes [38].

Both the taster and nontaster alleles have been preserved since the time of the Neanderthals [39] and so it is presumed that both alleles must do important work [22,40]. Humans would not have encountered PROP or PTC in their natural environment but structurally related compounds are found normally in some plants [41] and these chemicals are potent thyroid toxins [3]. Thus it is likely that the benefit of the taster allele is to detect thyroid poisons [42]. However, the function of the nontaster allele, if any, is not known [17]. One piece of evidence that supports the hypothesis that the nontaster form has a function is that it has an intact reading frame and is not a pseudogene [11] and therefore it might be able to detect bitter molecules from a different chemical class [43]. Another point to consider is that taste receptors are also found in the gut and stimulate hormone secretion [44] and their expression is dependent on diet [44]. Bearing these ideas in mind, perhaps there is an evolutionary advantage to being heterozygous: the taster allele is dominant early in life when thyroid poisons can do the most harm while the nontaster allele (and its unknown function) is more dominant during adulthood.

The above hypothesis suggests that the age and genotype effects are specific to one particular bitter receptor but there is another possibility. Instead, we hypothesize that age may modify the genotype-phenotype relationship for other sensory receptors. Studies of olfactory genes in the mouse demonstrate that the pattern of gene expression changes during development, with receptor genes turning off forever early in life and others only turning on in adulthood [45]. Olfactory gene expression changes during development, so perhaps individual alleles might do so as well. Some evidence exists which is consistent with this hypothesis. Similar to the present finding with PROP perception, there are age-related declines in olfactory sensitivity for the musk odor androstenone, a change which occur around the time of puberty [46]. Alleles of a particular smell receptor predict the threshold to this odorant [47]. While it is not known if the decline in androstenone sensitivity is more common in heterozygotes, if this were the case, it would suggest that development by genotype effects may be a feature of other sensory receptors.

Although previous studies have found that females taste PROP and chemically related compounds at lower thresholds than males [6], we observed no sex effects in the children and adolescents studied here. This result is probably explained by sexual maturation, because half of our subjects were children and sex effects for this trait are not typically found until after puberty [6] and the adults were almost all women. Regarding race/ethnicity, allele frequencies for the *TAS2R38 *gene vary by racial/ethnic group and therefore when two racial groups are compared, they differ in phenotype because they differ in genotype [18]. The differences in allele frequencies we found replicated those reported by others [11]. However we also asked a different question, which was whether race/ethnicity affects bitter threshold independent of genotype and here the answer was no. African American and Caucasian subjects with the same diplotype had indistinguishable bitter thresholds. Therefore differences in culture, experience and genetic background that are associated with race and ethnicity do not appear to modify this genotype-phenotype relationship.

Appropriate methods are critical in obtaining valid and reliable results when a wide age range of subjects is studied, so the rationale for the methods used herein should be considered. Bitter sensitivity to PROP and PTC have been assessed in the past in two ways (threshold versus intensity) and each method has several variations [26,48-50]. In this study, all subjects, regardless of age, were phenotyped the same way. A forced-choice categorization procedure enabled us to measure the lowest concentration at which they could recognize bitterness (threshold) rather than having them rate intensity. This method was chosen because children have difficulty with intensity measures and therefore threshold methods are preferable. This particular threshold method was developed by Anliker et al., (1994) and modified by Mennella et al., (2005) to specifically address several issues related to conducting research in pediatric populations, as follows: First, age-appropriate tasks which were fun and minimized the impact of language and cognitive development were used because young children are more prone to attention lapses and have shorter memory spans. Second, a forced-choice categorization procedure circumvents the element of uncertainty when tasting solutions at low concentrations. Furthermore, this method does not rely on 'yes' or 'no' answers which are prone to inaccuracy because young children tend to answer in the affirmative. Third, prior to the data collection and after a period of acclimation, we ascertained whether the child comprehended the task. Fourth, the same method was used for children, adolescents and adults so that any age-related differences observed were not due to disparity in the testing procedures. Albeit simple, this method proved to be reliable for children, adolescents and adults because subjects who were retested later demonstrate similar thresholds [32].

Children differ from adults since their likes and dislikes are the complex product of developing sensory systems, genetic variation, experiences and culture. In fact, they live in different worlds than adults in many sensory realms: sounds [51], smells [25,46], tastes [52] and irritants [53], but these differences are especially striking for bitter taste. In childhood, bitter sensitivity makes evolutionary sense because of the risk of accidental poisoning while foraging for plant foods, some of which may be poisonous [54]. But in the modern world, bitter sensitivity may take a toll on nutrition and health. An important example is that wholesome foods like vegetables are initially rejected, and this reluctance can later develop into a permanent avoidance because the positive aspects of the food are not experienced. Children need to be given repeated opportunities to learn to like bitter-tasting vegetables [55], maybe more so if they have bitter sensitive genotypes and the reluctance of parents and caregivers to offer foods that are initially rejected must be overcome [55]. Likewise, liquid formulations of medicines are avoided or refused by children because of their repellent taste [56,57]. Thus the rejection of unpalatable medications and bitter-tasting foods by children is a reflection of their basic biology and at least for vegetables can be fine-tuned by learning and genotype. A better understanding of the individual differences in chemosensory perception throughout the lifespan and the scientific basis for distaste and how to ameliorate it is a public health priority [56].

## Conclusion

Our data suggest that bitter sensitivity for at least one stimulus, PROP, changes over the lifespan and is affected by the person's genotype for alleles with the bitter receptor *TAS2R38*. These developmental sensory changes are most marked for people who have a particular haplotype combination, i.e., AVI/PAV heterozygotes.

## Method

### Phenotyping for PROP perception

To measure PROP perception, we used previously validated procedures that are sensitive to the cognitive limitations of pediatric populations [32]. Following a one-hour fast, each subject was tested individually in a closed room designed for sensory studies. Most of the children younger than 7 years were tested with their mothers present. The mothers, who sat behind the children and out of view, refrained from talking during the test session and listened to music with headphones to prevent them from hearing their children's answers. All other subjects were tested individually.

To allow for comparisons between the age groups, procedures were identical for all subjects and several steps were undertaken to make sure that the younger subjects understood the task before testing. The forced-choice procedures and concentrations of PROP used were based on previous research [24,32]. Subjects were presented with a cup containing 5 milliliters of water and told to rinse the contents in their mouth and then spit it out. If the solution tasted like water, they were told to give it to a stuffed toy of Big Bird™ (a likeable, well-known television character puppet), but if it tasted "yucky" or bitter, they should give it to Oscar the Grouch,™ so that he can "throw it in his trash can" [58]. The procedure was repeated and subjects tasted (but did not swallow), in ascending order, three solutions of PROP (56, 180 and 560 μM) rinsing with water before and after each tasting. (If a subject swallowed the PROP solution, the testing was immediately discontinued). Subjects were classified into four groups based on the lowest concentration, if any, that they reported bitterness and, in turn, gave the sample to Oscar the Grouch. Those who gave all samples to Big Bird were classified as "None (of the samples) tasted bitter".

To determine the reliability of the method, PROP testing was conducted a second time on a random sample of 34 children and 22 adults. Testing occurred 9.2 ± 1.3 months after the initial test session. Reliability, which was assessed by conducting *Kendall's tau *(*T*) correlations, revealed that the grouping based on the PROP threshold data obtained during the initial session was significantly correlated with that obtained during the retest for both children (*Kendall's T *= 0.69; p < 0.05) and adults (*Kendall's T *= 0.76; p < 0.05). Twenty four of the children (71%) and 15 of the adults (68%) reported first perceiving a bitter taste at the same concentration as originally reported. Of the remaining 10 children, 8 reported a concentration one step below and 2 reported a concentration one step higher during the second test. Of the remaining 7 adults, 4 had thresholds one step below and 1 had a threshold two steps below the original.

### Genotyping and haplotyping for *TAS2R38 *gene

Cells from the cheek were obtained using swabs and genomic DNA was extracted following the directions of the manufacturer (Epicenter, Madison, WI). Alleles of the *TAS2R38 *gene (Genbank accession no. NM_176817) were genotyped using real time PCR single nucleotide polymorphism (SNP) genotyping assays (*rs713598*, *rs1726866 *and *rs10246939*) with the Prism 7000, manufactured by Applied Biosystems (Foster City, CA). Haplotypes were identified by tracing the parental origin of the alleles when possible, or otherwise they were inferred by expectation-maximization methods using an algorithm implemented by the computer program fastPHASE [59].

### Data analyses

We conducted a multinomial logistic regression analysis with three most common diplotypes (AVI/AVI, AVI/PAV and PAV/PAV) and three age categories (children, adolescents, adults) as fixed factors and with a four-category outcome measure (subjects who first reported 56 uM, 180 uM or 560 uM PROP as bitter or subjects who reported that none of the solutions tasted bitter). The model was as follows: outcome measure = diplotype + age + diplotype × age. Analyses were re conducted after randomly removing heterozygous subjects to equate group sizes. If a significant interaction between diplotype and age were obtained, other methods outlined below were conducted to determine the nature of the interactions.

Most analyses followed the same method, which was to stratify subjects into groups by the variable of interest, and test for differences between the groups with an omnibus χ^2 ^analyses for *k *independent samples, followed by a partitioned χ^2 ^to determine where the difference occurred [60]. This analysis cannot be undertaken if there are no observations in a particular cell, or when the number of observations in 20% of the cells is less than five, so in some cases, we conducted analysis only on groups with sufficient sample size, or we combined related groups to increase the sample size per cell. (These conditions occasionally required that we do a test between two proportions rather than a χ^2^). To determine the effects of age, subjects were stratified by age and diplotype and the percentages of children, adolescents and adults who could taste PROP at each concentration were compared. We tested for race/ethnicity effects on PROP thresholds within particular diplotypes. For instance, we determined whether African-American subjects with the PAV/PAV diplotype had lower PROP thresholds than did Caucasians with the same diplotype. Subjects were also grouped by sex, to determine whether boys or girls differed in PROP thresholds, or by race/ethnicity, to determine whether these groups differed in haplotype frequency. Descriptive analyses, multinomial logistic regression and the proportion test were conducted with procedures in STATISTICA (StatSoft, Tulsa OK) and χ2 partitioning was calculated using a program described by Siegel and Castellan [60]. Criterion for statistical significance for all analyses was p ≤ 0.05.

## Abbreviations

PTC: phenylthiocarbamide; PROP: propylthiouracil

## Authors' contributions

JAM designed, supervised data collection, supervised staff, analyzed data and wrote the manuscript. MYP collected and analyzed the data and assisted in manuscript preparation. FD collected and analyzed data and assisted in manuscript preparation. DRR supervised genotype data collection and co-wrote the manuscript. All authors read and approved the final manuscript.

## Authors' information

This work was supported by the National Institutes of Health [HD37119, AA09523 to J.A.M. and DC004698 to D.R.R.)]; and a grant from the Pennsylvania Department of Health. The Pennsylvania Department of Health specifically disclaims responsibility for any analyses, interpretations, or conclusions. Dr. Pepino is currently a fellow through a National Institute of Drug Abuse training grant administered by the School of Medicine, Washington University in St. Louis, MO (#T32 DA07313).
